# Darwin, the devil, and the management of transmissible cancers

**DOI:** 10.1111/cobi.13644

**Published:** 2020-10-08

**Authors:** Rodrigo Hamede, Thomas Madsen, Hamish McCallum, Andrew Storfer, Paul A. Hohenlohe, Hannah Siddle, Jim Kaufman, Mathieu Giraudeau, Menna Jones, Frédéric Thomas, Beata Ujvari

**Affiliations:** ^1^ School of Natural Sciences University of Tasmania Hobart Tasmania Australia; ^2^ School of Biological Sciences University of Wollongong Wollongong New South Wales Australia; ^3^ School of Environment and Science Griffith University, Nathan Campus Nathan Queensland Australia; ^4^ School of Biological Sciences Washington State University Pullman WA U.S.A.; ^5^ Department of Biological Sciences Institute for Bioinformatics and Evolutionary Studies, University of Idaho Moscow ID U.S.A.; ^6^ Centre for Biological Sciences University of Southampton Southampton SO17 1BJ U.K.; ^7^ Department of Pathology University of Cambridge Cambridge CB2 1QP U.K.; ^8^ Centre de Recherches Ecologiques et Evolutives sur le Cancer/Centre National de la Recherche Scientifique Montpellier France; ^9^ Centre for Integrative Ecology, School of Life and Environmental Sciences Deakin University Waurn Ponds Victoria Australia

## Introduction

Modern conservation science frequently relies on genetic tools to manage imperiled populations threatened by processes such as habitat fragmentation and infectious diseases. Translocation of individuals to restore genetic diversity (genetic rescue) is increasingly used to manage vulnerable populations (Whiteley et al. 2015), but it can swamp local adaptations and lead to outbreeding depression (Frankham et al. 2011). Thus, genetic management is context dependent and needs evaluation across multiple generations (Fitzpatrick et al. 2020). Genomic studies can help evaluate the extent to which populations are locally adapted to assess the costs and benefits of translocations. Predicting the long‐term fitness effects of genetic interventions and their evolutionary consequences is a vital step in managing dwindling populations threatened by emerging infectious diseases.

Multicellular organisms have a long evolutionary history with oncogenic phenomena. While some cancer‐protection adaptations are very old and phylogenetically well conserved, such as cancer‐suppression mechanisms (Nunney 2013), others are species specific and shaped by ecological processes and life‐history traits (Ujvari et al. 2016). An increasing number of infectious cancers, virus associated and directly transmissible, are occurring in terrestrial and aquatic environments (Hamede et al. 2020). Thus, cancer is nowadays regarded as a disease of conservation concern (McAloose & Newton 2009), particularly for threatened wildlife (Hamede at al. 2020). We considered the Tasmanian devil (*Sarcophilus harrisii*) and its transmissible cancers as a model to examine the integration of knowledge of host‐pathogen evolutionary interactions with wildlife disease management.

Devils have been afflicted by a transmissible cancer (devil facial tumor disease [DFTD]) for at least 24 years (Hawkins et al. 2006). The DFTD epidemic has caused significant population declines (McCallum et al. 2009) and led to the species’ listing as endangered (Hawkins et al. 2009). In 2014 another, independently evolved, transmissible cancer (devil facial tumor 2 [DFT2]) was discovered in southeastern Tasmania (Pye et al. 2016*b*).

Translocations of captive or free‐range devils sourced from insurance populations with the aim of genetic rescue (Grueber et al. 2019) are being tested, as are field immunizations aimed at stimulating an adaptive immune response (Pye et al. 2018). However, the epidemiological and evolutionary consequences of introducing naïve individuals from insurance populations into diseased populations have not been evaluated comprehensively, thus, current management is unlikely to prevent transmission. Darwinian principles, host‐pathogen coevolutionary theory, and the growing literature on ecological and evolutionary principles in oncology (Korolev et al. 2014) suggest that silver bullets are unlikely to result in disease eradication. We considered evolutionary biology and ecology of host‐pathogen interactions to highlight why the role of natural selection in host adaptations to cancer should be considered in the management of this species and other epizootics.

### Adaptations to DFTD in Wild Devils

**Figure 1:**
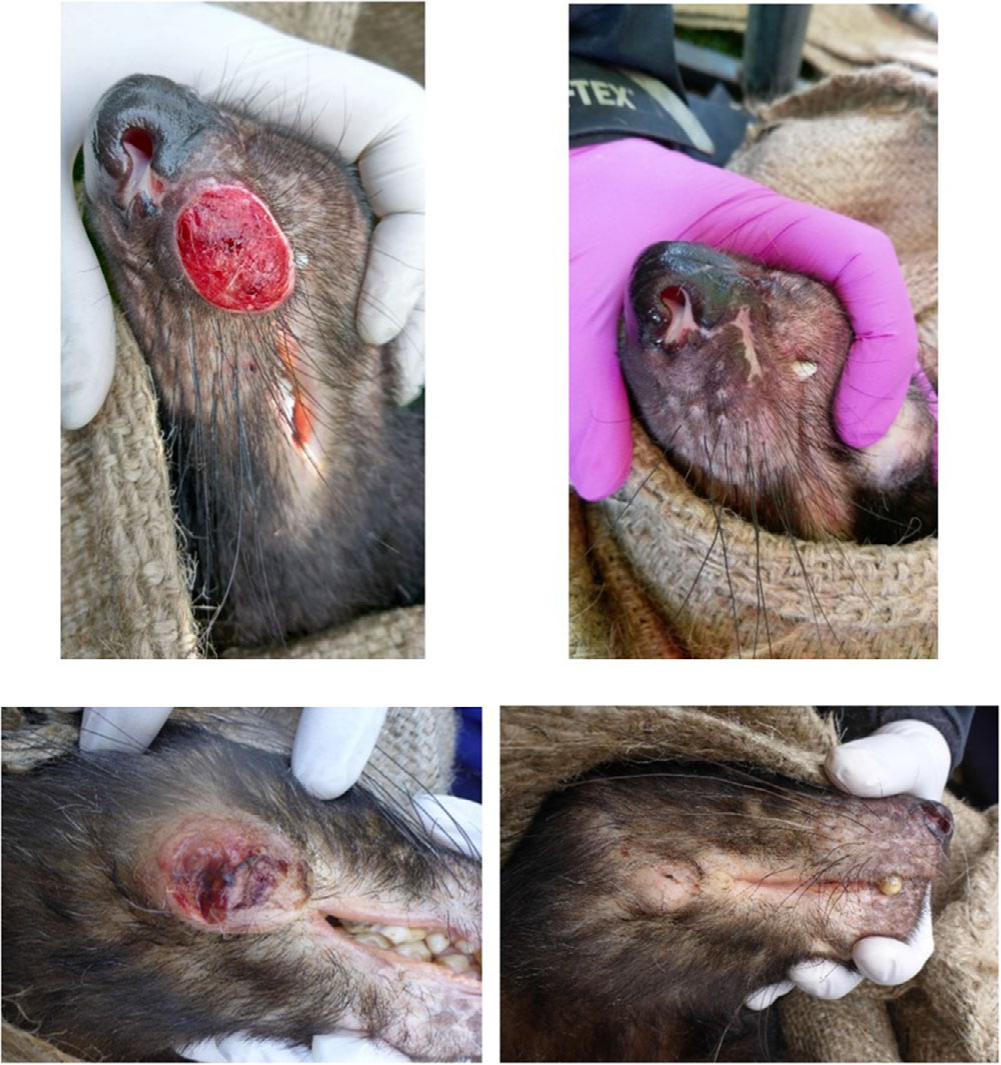
Two wild Tasmanian devils with DFTD (left) and after natural tumor regression 3 months later (right). Photos by Manuel Ruiz Aravena.

Early in the epizootic, DFTD caused localized population declines of up to 90% (McCallum et al. 2009). Once tumors were detected, they were universally fatal. After 10 years, DFTD had reduced effects at the epidemic frontline (Hamede et al. 2012). Devils from populations in northwestern Tasmania mounted immune responses to DFTD, concomitant with natural tumor regressions and recovery after infection (Pye et al. 2016*a*) (Fig 1). Devils with regressed tumors differed genetically from those with nonregressed tumors (Margres et al. 2018*a*), and tumor regressions appear to be affected by upregulation of putative tumor suppressor RASL11a, a gene not expressed in human cancers (Margres et al. 2020). Allele frequencies in genomic regions associated with immune function and cancer changed significantly 4‐6 generations after disease arrival (Epstein et al. 2016). These changes occurred in 3 geographically separated populations and concurred with patterns of cessation of population decline at these sites. Moreover, a small number of loci explains significant variation in case control (diseased vs. never diseased devils) and survival of infected females (Margres et al. 2018b). These results provide evidence of rapid evolutionary responses, likely resulting from extreme selection imposed by DFTD, and demonstrate that devils have sufficient genetic variation to respond and adapt to the DFTD epidemic, despite limited genetic diversity (Bruniche‐Olsen et al. 2014).

### Applying Evolutionary Principles to Disease Management and Devil Conservation

Evidence of devil evolutionary responses to DFTD in geographically widespread locations (east, north, and northwest) warrants a conservation strategy that facilitates these beneficial adaptations. However, current plans include translocating individuals sourced from captive, DFTD‐naïve populations into wild populations that have undergone generations of selection for resistance or tolerance. Increasing the number of susceptible individuals in diseased devil populations is likely to fuel the epidemic by increasing infection rates and potentially diluting or delaying selective processes. The adaptive response of the devil to DFTD involves interacting mechanisms and genes; thus, we suggest captive‐breeding programs incorporate wild‐sourced devils with genotypes that exhibit resistance or tolerance to DFTD. Future studies should identify evolutionary trade‐offs associated with resistance or tolerance to DFTD and their potential fitness effects.

To acquire herd immunity, over two‐thirds of a population would need to be vaccinated, given the estimated reproductive rate of DFTD (McCallum et al. 2009). Offspring of vaccinated devils would be susceptible and facilitate DFTD transmission. Although the development of a vaccine is laudable, with potential future benefits for management (Patchett & Woods 2019), premature vaccination in the wild could have detrimental effects, particularly if the vaccine cannot provide life‐time immunity in 1 shot. Vaccines that let the host survive and do not entirely prevent infection and spread can create ecological and epidemiological conditions that facilitate evolution and persistence of more virulent pathogen strains (e.g., Gandon et al. 2001; Mackinnon et al. 2008). Natural selection should remove highly lethal pathogens if the death rate of hosts significantly reduces transmission. However, if vaccinations prolong host survival without precluding transmission, pathogen replication is favored (Read et al. 2015), which may result in higher prevalence and favor persistence of tumor strains that might not be viable under natural selection. Therefore, before widespread vaccination in the wild, experiments to prove the prophylactic effect of vaccines and modeling of epidemic outcomes should be conducted.

Coexistence of devils and DFTD is the most likely long‐term enzootic outcome predicted by epidemiological models based solely on ecological processes (i.e., no explicit genetic component) (Wells et al. 2019). Incorporating evolutionary change in epidemiological models will likely strengthen the likelihood of coexistence of devils and DFTD without management. However, incorporating loss of genetic diversity may decrease the likelihood of coexistence without further intervention. One of the greatest difficulties, characterized by emerging infectious diseases and anthropogenic disturbance, is to determine whether human intervention should be undertaken and whether its benefits outweigh the benefits of facilitating recovery through natural selection. Interventions that enhance the prospects of devil recovery via local adaptation should be prioritized, but relying solely on evolution of natural adaptations to protect the species is risky, particularly considering that the evolutionary interactions and epidemiological outcomes between DFTD and DFT2 have not been evaluated fully. The emergent DFT2 warrants further caution because observed adaptations to DFTD may not be relevant to DFT2. The cumulative effects of 2 transmissible cancers and other threats need to be considered to balance the fitness effects of locally adapted populations to transmissible tumors and the loss of genetic diversity.

There is a fundamental conundrum between maximizing genetic diversity (genetic rescue) and allowing natural selection to progress (evolutionary rescue). In the case of devils, where much empirical evidence shows rapid evolution in response to DFTD in the wild and potential functional genetic changes have been identified, we argue that management of adaptive genetic diversity should be prioritized.

Captive‐breeding programs to maintain genetic diversity are fundamental to protecting vulnerable populations. The case of Tasmanian devils and their cancer epizootics illustrates the importance of developing cross‐disciplinary approaches capable of timely assessments of the ecoevolutionary processes between hosts and pathogens and their consideration for management. Therefore, we encourage managers and researchers to engage in a scientific debate that integrates new knowledge and offers novel methods to assess the effects of management on evolutionary and epidemiological dynamics in the devil‐DFTD‐DFT2 system.

Evolutionary responses to pathogens with high mortality are not restricted to devils and DFTD. There is evidence of rapid evolutionary change in other host populations subject to emerging wildlife diseases (e.g., frogs in Central America exposed to chytrid fungus [Voyles et al. 2018]). Principles of evolutionary versus genetic rescue are also relevant in other contexts, such as rapid environmental change (e.g., climate‐adjusted provenancing), and have direct implications for biodiversity conservation and ecological restoration (Prober et al. 2015). Genomic research is fine tuning and improving the capacity to understand the genetic basis of adaptation. Balancing the priorities of genetic rescue and evolutionary rescue is a critical task for conservation. Modern genomic methods, as exemplified by work on Tasmanian devils and DFTD, provide new tools for integrated management of populations threatened by diseases. For researchers and managers, the fundamental principle should be as espoused in the Hippocratic Oath, *first, do no harm*.
